# Clinical and molecular genetic analysis of a Chinese patient with Cockayne syndrome caused by *ERCC8* gene synonymous variant at splicing site and exon 1 deletion

**DOI:** 10.1186/s13023-025-03808-y

**Published:** 2025-11-26

**Authors:** Xiaofan Bie, Lei Liu, Lingzhi Liu, Zhenkun Zhang, Mingwei Guo, Zhenhua Xie, Yaodong Zhang, Jun Su, Dongxiao Li

**Affiliations:** 1 Henan Children’s Neurodevelopment Engineering Research Center, Children’s Hospital Affiliated to Zhengzhou University, Henan Children’s Hospital Zhengzhou Children’s Hospital, Zhengzhou, China; 2 Pediatric Intensive Care Unit of Henan Children ’s Hospital, Children’s Hospital Affiliated to Zhengzhou University, Henan Children’s Hospital Zhengzhou Children’s Hospital, Zhengzhou, China

**Keywords:** Cockayne syndrome, *ERCC8*, Splice site, Synonymous variation

## Abstract

**Background:**

Cockayne Syndrome (CS) is a rare autosomal recessive genetic disease, mainly caused by *ERCC8* and *ERCC6* gene defect. However, many of its molecular characteristics remain unclear. In this study, molecular genetic analysis was performed on a patient to clarify her genetic etiology.

**Results:**

A 7-year-old girl fever for 4 days and thrombocytopenia for half a day. Her main clinical manifestations included lethargy after infection, short stature, microcephaly, mental retardation, facial aging, skin photosensitivity. Laboratory tests indicated liver and kidney damage, thrombocytopenia, and brain MRI revealed progressive brain damage. Whole exome sequencing showed that the proband had a c.1041G > A (p. Gln347=) heterozygous synonymous variation and a suspected large fragment heterozygous deletion containing exon 1 of *ERCC8* gene. Sanger sequencing and Quantitative real-time PCR were respectively used to confirm inheritance from her phenotypically normal mother and father. Transcriptome sequencing showed a deletion of exon 10. According to the ACMG guidelines, the two variations were classified as pathogenic variants.

**Conclusions:**

This study reported the rare case of CS caused by the c.1041G > A synonymous variation causing exon 10 deletion by affecting splicing and large fragment deletion containing exon 1 by preventing its allele from initiating transcription, expanding the variation spectrum of the ERCC8 gene. It reminds us that although synonymous variations are rare, they may affect splicing when they occur at the junction of exons and introns.

## Introduction

Cockayne syndrome (CS) is a rare autosomal recessive disorder characterized by short stature, microcephaly, developmental delay, growth retardation, premature aging and photosensitivity. It is often accompanied by multi-system damage [11, 16]. The CS phenotypes encompass a continuous and overlapping spectrum, including brain-eye-face-bone syndrome (COFS), Cockayne syndrome type I, II, III, and ultraviolet sensitive syndrome [7, 9]. About 1/3 of CS patients exhibit *ERCC8* gene (OMIM*609412) deficiency, while about two-thirds have *ERCC6* gene (OMIM*609413) deficiency. These genes were identified as the pathogenic genes of CS in 1992 and 1995, respectively [2, 6, 12].

The clinical symptoms and severity of CS patients are highly heterogeneous, making accurate prediction at the stage of diagnosis challenging [2]. CS lacks specific biochemical or metabolic markers, with clinical diagnosis relying primarily on clinical manifestations and imaging features, and further confirmed by molecular genetic testing [10]. Most reported *ERCC8* gene variations were frameshift and missense, with no documented cases of CS caused by synonymous variations.

In this study, we reported a 7-year-old girl with CS caused by synonymous variation of *ERCC8* gene and described the gradual progression of her brain condition, expanding the variation spectrum of *ERCC8* gene and providing new insights for CS diagnosis.

## Materials and methods

### Patient

The clinical data and test results of the proband were collected, including blood and urine routine tests, liver and kidney function, myocardial enzyme, electrolyte, thyroid function, X-ray, color doppler ultrasound, and brain MRI. With approval from the Ethics Committee of Henan Children’s Hospital and informed consent from the patient’s family, 3 ml of peripheral venous blood was extracted from the patient and her parents. The genomic DNA of the patient and her family was extracted using the peripheral blood genomic DNA extraction kit (Tiangen, China).

### Whole exome sequencing

The whole exome sequencing was performed at the age of 3. DNA fragments of the target region were enriched by using IDT’s xGEN Exome Research Panel v2.0 capture probe to construct a whole exome library. The qualified libraries were sequenced by Illumina sequencer (Hiseq2000) for high-throughput sequencing. The raw sequencing data were compared with the reference genome GRCh37/hg19 by BWA bioinformatics software to remove low-quality variations. After passing Illumina Sequence Control Software quality checks, data reading and bioinformatics analysis were conducted. Data interpretation adhered to the guidelines provided by the American College of Medical Genetics and Genomics (ACMG). Variant nomenclature followed the rules suggested by the Human Genome Variation Society (http://www.HGVS.org/varnomen).

The whole exome sequencing data was reanalyzed at the age of 7, with a particular focus on the *ERCC6* and *ERCC8* genes.

### Bioinformatics analysis

Mutation Taster (https://www.mutationtaster.org/), CADD-Combined Annotation Dependent Depletion (CADD, https://cadd.gs.washington.edu/) and Splice Site Prediction (SSP, https://fruitfly.org/seq_tools/splice.html) online databases were used to predict and analyze the variation.

### Sanger sequencing to verify the variations

Primers were designed upstream and downstream of the *ERCC8* gene (NM_000082.3) c.1041 variation site, *ERCC8*-F1: 5’- GTGGCTGCAGTTCAGAATTTGTTT − 3’, *ERCC8*-R1:5’- ACAATCTAGGCTGTGCTATTCTCT − 3’. PCR amplification was performed using the KAPA2G Robµst HotStart PCR Kit (Roche), and the PCR product was further purified for Sanger sequencing.

### Quantitative real-time PCR

Hieff qPCR SYBR Green Master Mix Kit (Yeasen, China) was used for Quantitative real-time PCR validation of microdeletion variants. Primers was designed for exon 1 of *ERCC8* gene, *ERCC8*-F2: 5’-TTTGGCGTGCGGACAAAAACC-3’, *ERCC8*-R2:5’-GACTCTGCTGTTCCAGTCCCG-3’; Primers were also designed for the reference gene *ALB* exon 13, *ALB*-F:5-ACTCAGTGCACTTGTTGAGCTCGT-3’, *ALB* -R:5’-TCGTCAGCCTTGCAGCACTTCT-3’.

### RNA sequencing

TRnaZol Reagent kit (NCM Biotech, China) was used to extract the whole genome RNA from peripheral blood of proband and her parents. The RNA was reverse transcribed into cDNA using Hifair III 1st Strand cDNA Synthesis SuperMix for qPCR kit (Yeasen, China), and quantified by NanoDrop 2000 ultraviolet spectrophotometer (Thermo Fisher Scientific, USA).

### Sanger sequencing

The cDNA of *ERCC8* gene was used as a template to design primers upstream and downstream of c.1041 variation site, *ERCC8*-F3: 5’-CTCCAGTCTCCACCAAGCAC-3’, *ERCC8*-R3: 5’-GGCATCTTCAAAGGCCGGAT-3’, and targeted fragment was amplified. The *WDR45* gene, expressed in the blood, was selected as the reference gene due to its product length being similar to that of the *ERCC8* gene product. *WDR45*-F: 5’- CTCGTCTGCTCCATTCACGA-3’, *WDR45*-R:5’- CACGTCGAAAGCCTCTCTGT-3’. Subsequently, the PCR product was qualified by electrophoresis and Sanger sequencing was used to detect the possible cDNA changes caused by c.1041G > A variation.

### RNA high-throughput sequencing

The high-throughput sequencing platform was used to perform PE150 mode sequencing, and the expression levels of different RNAs were calculated by counting the number of related reads. The data were subjected to sequence alignment, differential expression analysis, gene function annotation and functional enrichment analysis by comparing with the designated reference genome. Additionally, we used rMATS 4.0.2 software to customize splicing analysis which was performed on chromosome 5, where ERCC8 gene is located.

## Results

### Clinical finding

The proband was a 7-year-old girl, G1P1. Born via full-term caesarean section with a birth weight of 3.25 kg. She was admitted to our emergency department with “fever for 4 days and thrombocytopenia for half a day”, mainly manifested as low spirit after infection, thrombocytopenia, abnormal liver and kidney function, pneumonia, pleural effusion and other multiple organ dysfunctions. The proband exhibited delayed physical and intellectual motor development since childhood. She raised her head at 4 months, sit independently at 8 months, crawl at 9 months, stand independently at 15 months, and walk independently at 1 year and 8 months. She has an abnormal walking posture, runs slowly, dresses and undresses herself, eats by herself, and holds her pen unsteadily. She has learning difficulties, can communicate daily but has a slow speech rate, and shows progressive dwarfism and microcephaly. She visited rehabilitation department at the age of 3 due to developmental delay.

Physical examination: the patient’s height was 98 cm (-5SD), weight was 13 kg (<-3SD), head circumference was 47.5 cm (<-2SD), and body temperature was 37.5 °C. The patient had consciousness, lethargy, emaciation, sunken eye socket and eagle-hooked nose. Muscle volume was reduced, limb muscle tension increased, bilateral knee tendon reflex was active, and bilateral Babinski sign were positive.

### Auxiliary examination

**Fig. 1:**
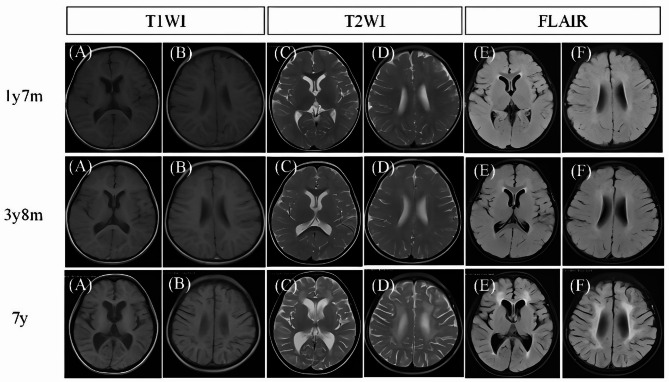
Brain magnetic resonance imaging (MRI) of the proband. At the age of 1 year and 7 months, no obvious abnormalities were detected. Brain MRI at 3 years and 8 months indicated decreased white matter volume and hypomyelination. At the age of 7 years, (**A**, **B**) Deepened sulci in the bilateral cerebral hemisphere. (**C**, **D**) T2WI showed significantly decreased white matter of bilateral cerebral hemispheres. (**E**, **F**) Patchy FLAIR high signals around the bilateral ventricles

Blood platelet count was 33 × 10^9^/L (normal range: 167–453 × 10^9^/L). Urine protein was 2+. Liver and kidney function tests showed: alanine aminotransferase 117.2 U/L (normal range: 7–30 U/L), urea nitrogen 21.05 mmol/L (normal range: 2.5–6.5 mmol/L), creatinine 124.9 µmol/L (normal range: 27–66 µmol/L), uric acid 466.3 µmol/L (normal range: 149–369 µmol/L). The 24-hour urinary protein quantitation was 8.611 g/24 h (normal range: 0.03–0.15 g/24 h). Thyroid function was normal. Abdominal and urinary ultrasound revealed hepatomegaly, slightly echogenic parenchyma, splenomegaly, and abdominal and pelvic effusion. Chest CT showed pneumonia, bilateral pleural effusion, right pneumothorax, right chest wall soft tissue swelling, and gas accumulation. Brain MRI at 1 year and 7 months showed no obvious abnormalities. Brain MRI at 3 years and 8 months indicated decreased white matter volume and hypomyelination compared to children of the same age. At 7 years old, brain MRI showed deepened sulci in the bilateral cerebral and cerebellar hemispheres, hypomyelination and decreased white matter volume (Fig. 1).

### Molecular genetic analysis

#### Whole exome sequencing and quantitative real-time PCR results

**Fig. 2:**
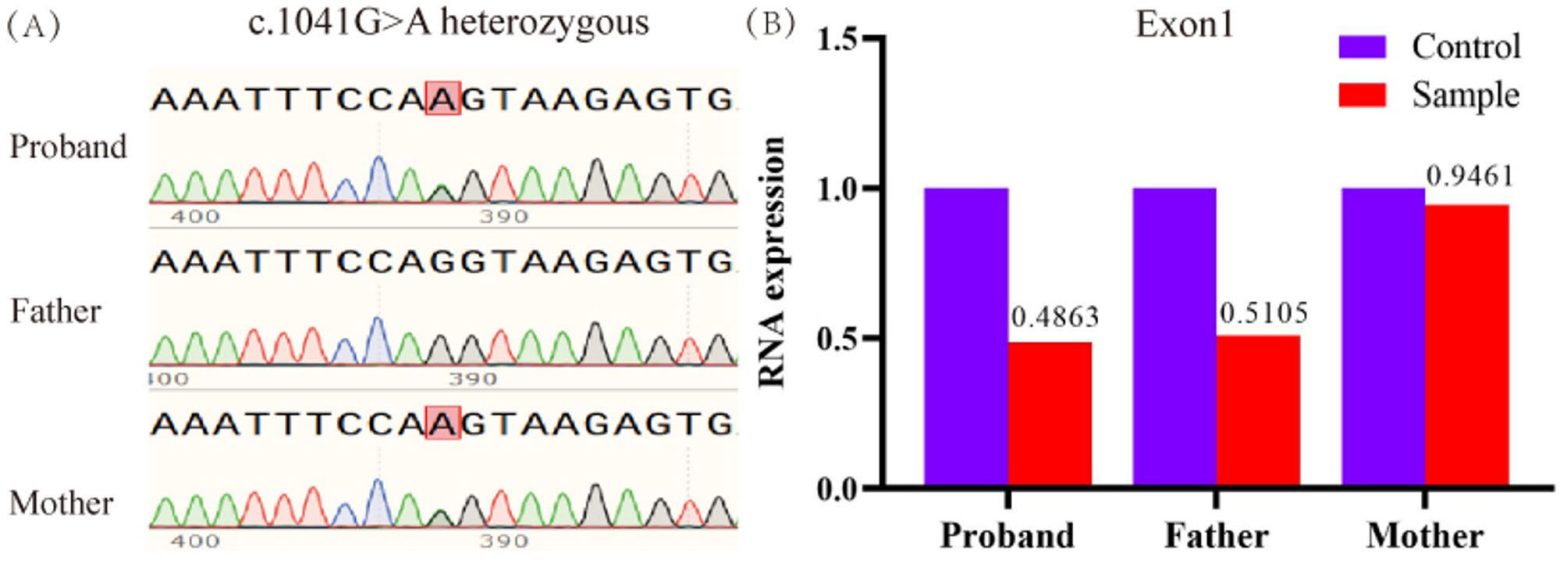
(**A**) Proband family sequencing. Sanger sequencing showed that the c.1041 locus of the proband and mother changed from G to A. (**B**) Quantitative real-time PCR results indicated that both the proband and her father exhibited exon 1 deletion

The whole exome sequencing performed at the age of 3 yielded negative results. However, reanalysis at the age of 7 revealed a suspicious biallelic variant in the *ERCC8* gene c.1041G> A (p. Gln347=) and a suspicious large fragment deletion containing exon 1. The c.1041G > A variant is located at the junction of exon 10 and intron 10. Sanger sequencing showed that c.1041G > A was inherited from the mother who has a normal phenotype (Fig. 2A). Quantitative real-time PCR confirmed that the proband had a deletion of exon 1, inherited from the father, who has a normal phenotype, while the mother was wild type at this site (Fig. 2B). The c.1041G > A variant was not found in the 1000 Genomes, HGMD, gnomAD, or ClinVar databases. Prediction tools Mutation taster (Disease_causing, predicted value of 1.0) and CADD software (predicted value of 33) indicated harmful effects, with an SSP score of 0.93. According to the ACMG guidelines, c.1041G > A (p. Gln347=) (PM2_supporting + PM3 + PP3 + PP4) was classified as a variant of uncertain significance, and large fragment deletion containing exon 1 of *ERCC8* gene (PVS1 + PM2_supporting + PP4) was classified as pathogenic.

### RNA amplification and sequencing

**Fig. 3:**
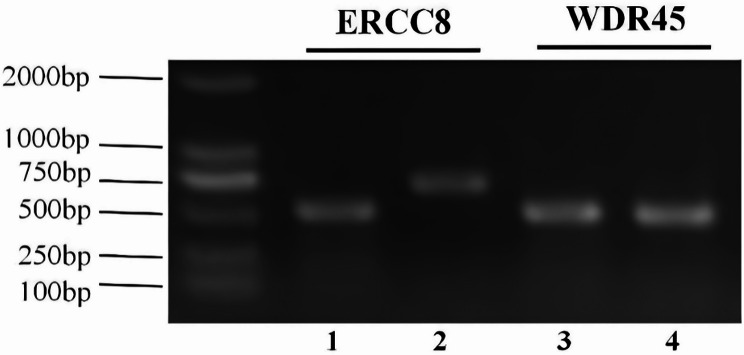
Expression of *ERCC8* mRNA in peripheral blood. The reference gene is *WDR45*. The target band of the *ERCC8* gene was amplified at 716 bp, and the target band of the *WDR45* gene was amplified at 508 bp. Samples 1 and 3 are from the proband, while samples 2 and 4 are normal controls. Results from samples 1 and 2 showed that the *ERCC8* gene in the patient’s peripheral blood was approximately 198 bp shorter than in the normal controls. Results from samples 3 and 4 showed normal expression of the *WDR45* gene in peripheral blood

**Fig. 4:**
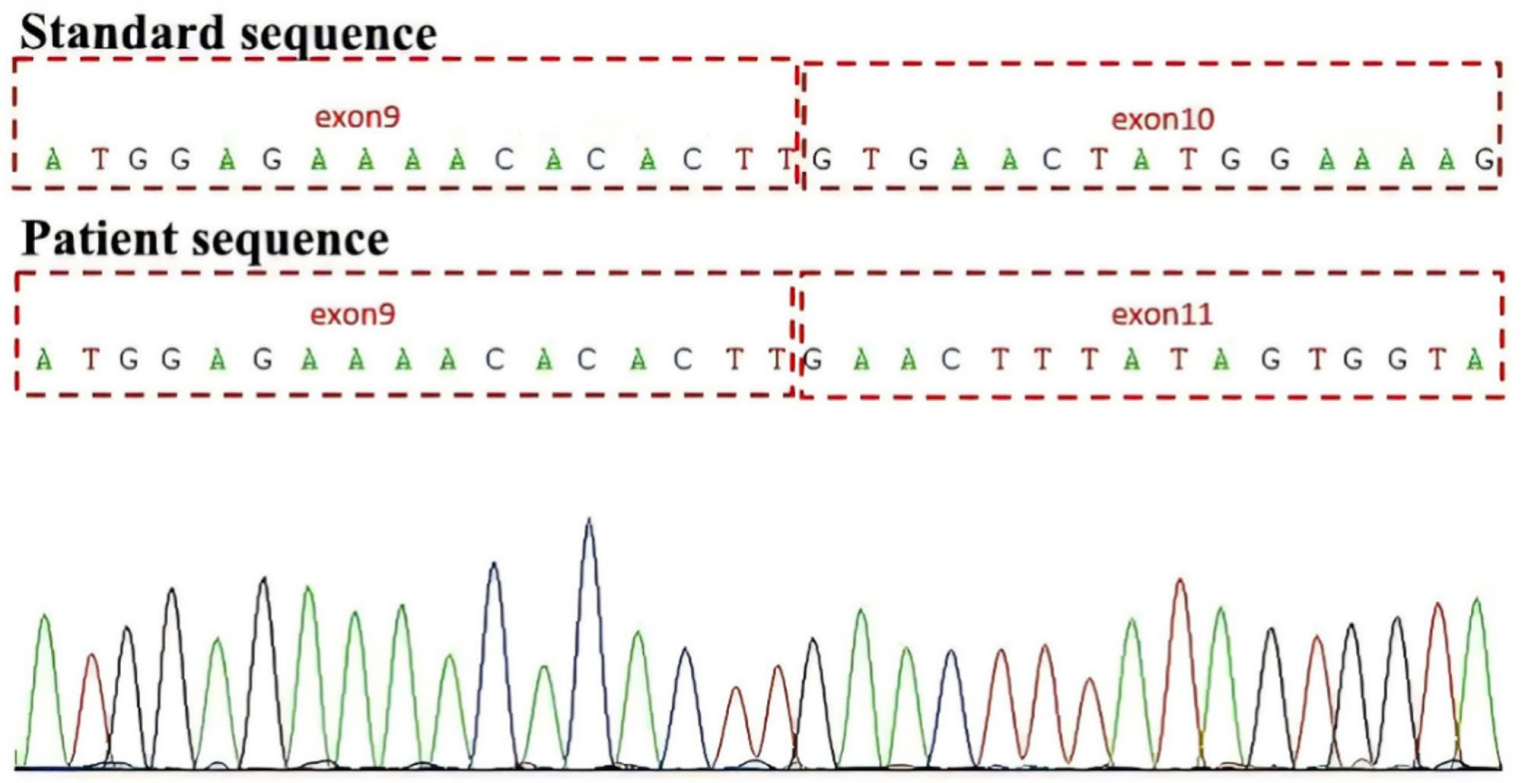
Sanger sequencing results of the patient. The patient’s Sanger sequencing results showed an exon 10 deletion relative to the standard sequence

**Fig. 5:**
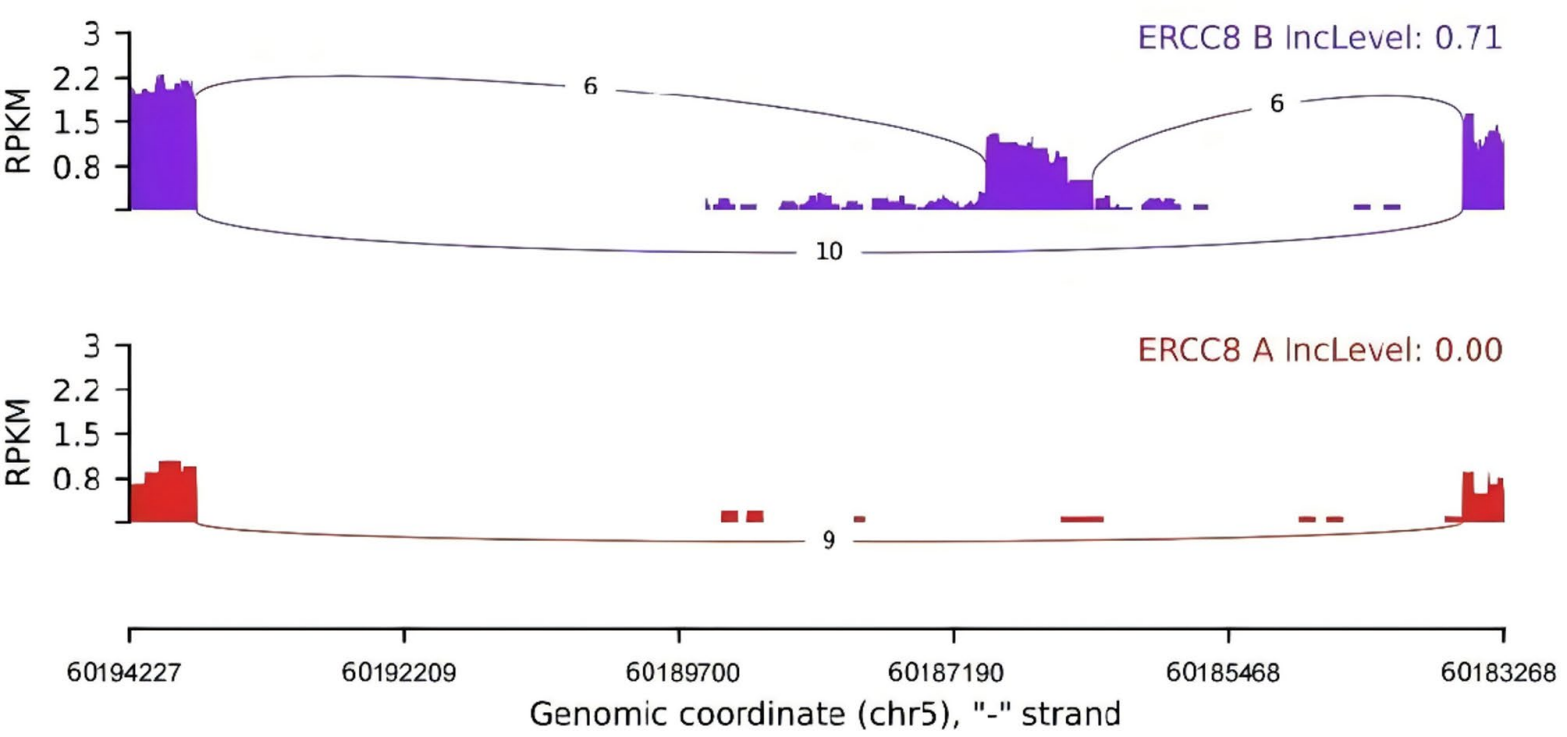
Exon 10 customized splicing analysis. Red A represents the proband, and purple B represents the parent. The lines indicate the number of reads. A higher number of reads signifies stronger evidence for the presence of exons. The results showed that, compared with the parent, the proband had an exon 10 deletion at the transcriptional level

PCR electrophoresis experiments showed that the length of *ERCC8* gene amplification in the proband was 518 bp, which was 198 bp shorter than the 716 bp product of the normal control sample, corresponding precisely to the length of exon 10 (198 bp) (Fig. 3). cDNA Sanger sequencing confirmed the exon 10 deletion (Fig. 4). RNA high-throughput sequencing results demonstrated that exon 10 was significantly absent at the transcriptional level (Fig. 5). According to ACMG guidelines, c.1041G > A (p.282_347del) (PVS1 + PM2_supporting + PM3 + PP4) is classified as a pathogenic variant.

**Fig. 6:**
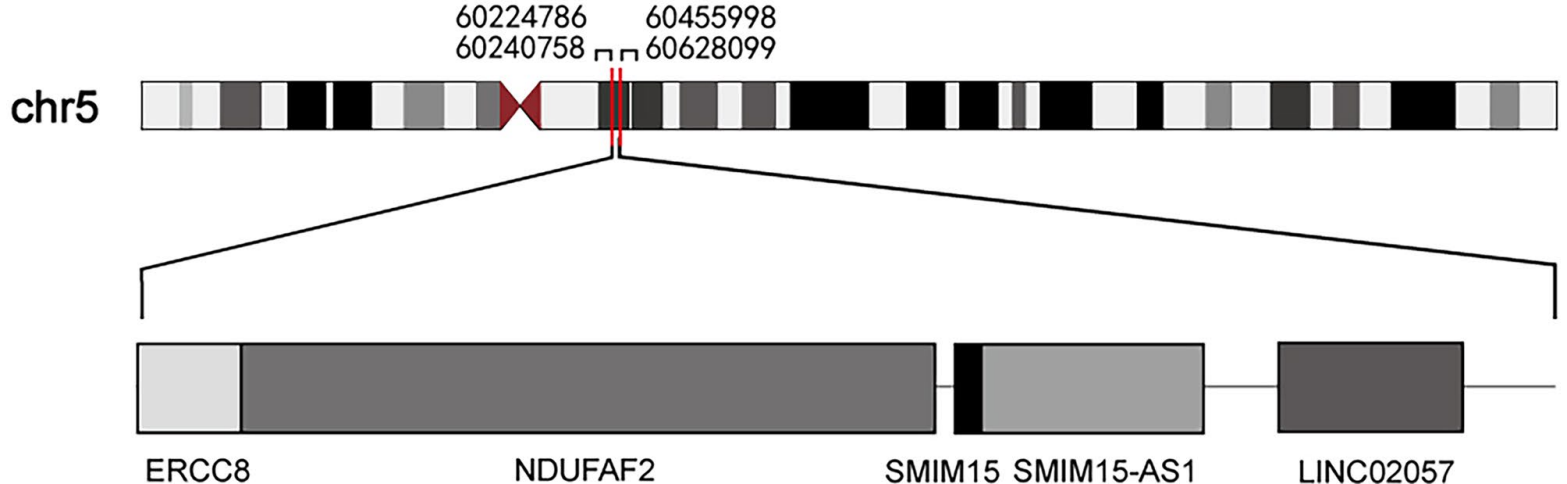
Genes on chr5:60224786–60,628,099. The genes located between chr5: 60,224,786 and chr5: 60,628,099 are *ERCC8*, *NDUFAF2*, *SMIM15*, *SMIM15-AS1* and *LINC02057*

Additionally, the *NDUFAF2* gene and *SMIM15* gene are located in close proximity to the *ERCC8* gene (Fig. 6). By analyzing the raw transcriptome data of individual genes, differentially expressed genes (DEGs) were identified based on the criteria of Fold Change ≥ 2 and FDR < 0.01. Under these conditions, the *NDUFAF2* gene and *SMIM15* gene exhibited reduced expression in the proband and her father compared to the mother, who had a normal exon 1 of *ERCC8* gene and served as the control. The expression of *ZSWIM6* gene returned to normal, leading to the conclusion that a large fragment deletion occurred from intron 1of *ERCC8* gene to the *ZSWIM6* gene.

Therefore, two breakpoint intervals of the deletion are speculated, the upstream breakpoint is between chr5: 60,224,786 and chr5: 60,240,758, and the downstream breakpoint is between chr5: 60,455,998 and chr5: 60,628,099.

## Discussion

The prevalence of CS in Japan is 2.5 per million [16]. Although the incidence of the disease is low, the prognosis is poor and there is no medicine to treat or cure it. Symptomatic treatment and avoidance of sunlight exposure are the only ways to prolong life. Studies have shown that the variation spectrum of *ERCC8* in Chinese is different from that in other populations. Exon 4 rearrangement is the main variation in CSA patients of Chinese Han nationality [14]. To date, the HGMD Professional database has revealed 96 variations in the *ERCC8* gene, missense/nonsense variations (39.6%), splicing variations (22.9%), small deletions (16.7%), small insertions (5.2%), small indels (3.1%), gross deletions (10.4%) and complex rearrangements (2.1%). The clinical symptoms of patients with nonsense and frameshift variations may be more typical, while the clinical symptoms of patients with missense variations are less pronounced. These results suggest the heterogeneity of CS phenotype [15].

*ERCC8* is located on 5q12.1 and consists of 12 exons, encoding a CSA protein containing 396 amino acids. The protein contains seven highly conserved repeat WD40 domain motifs (short ~ 40 amino acids residues often terminating in a Trp-Asp (W-D) dipeptide) [6]. The CSA protein mediates an important DNA repair pathway, nucleotide excision repair (NER), a DNA repair system present in a various species to remove DNA damage. NER consists of global genome NER (GGNER) and transcription-coupled NER (TCNER). TCNER is initiated after RNA Pol II recognizes transcriptional damage, which not only effectively prevent transcription at the damaged site but also recruits *ERCC8* and *ERCC6*. Deficiency in these two genes can induce the occurrence of CS and UV-sensitive syndrome [3, 4, 12].

In this study, the proband exhibited a chronic onset type with gradual progression, showing typical clinical manifestations of CS. Laboratory tests revealed multiple organ damage, brain atrophy, and decreased white matter volume, clinically consistent with Cockayne syndrome. At the age of 3, she underwent whole exome sequencing for a diagnosis of “developmental delay,” but the results were negative. The unusual phenotype and limited phenotype keywords made it challenging to identify pathogenic variants. Additionally, filtering out synonymous variants and suspicious single exon deletion contributed to the negative outcome of the whole exome sequencing.

To confirm the diagnosis, further genetic testing was performed. Whole exome sequencing revealed that the proband carried the *ERCC8* gene c.1041G > A (p. Gln347=) heterozygous variation and a suspected large fragment heterozygous deletion containing exon 1 of *ERCC8* gene. Sanger sequencing showed that the heterozygous variation of c.1041G> A was inherited from the mother, who had a normal phenotype, and was an unreported variation. Quantitative real-time PCR was used to rule out the possibility of false positives for the exon 1 deletion and confirmed that the variation came from a father with a normal phenotype, consistent with the autosomal recessive inheritance model. According to the ACMG guidelines, c.1041G > A (PM2_supporting + PM3 + PP3 + PP4) was rated as a variant of uncertain significance, while the large fragment deletion containing exon 1 (PVS1 + PM2_supporting + PP4) was classified as a pathogenic variation.

Decreased expression of both *NDUFAF2* and *SMIM15* genes in transcriptome data suggests that the large fragment deletion containing exon 1 of *ERCC8* gene may affect the transcription of both the *NDUFAF2* and *SMIM15* genes. Examing the genes involved in this deletion range, the *NDUFAF2* gene causes Mitochondrial complex I deficiency, nuclear type 10. However, since the *NDUFAF2* gene follows an autosomal recessive inheritance pattern, both the patient and her father are likely carriers without exhibiting the disease. The *SMIM15* gene phenotype is not listed in OMIM, and no diseases have been reported to be associated with the *SMIM15* gene. *SMIM15-AS1* and *LINC02057* genes are non-coding RNA that do not encode proteins.

The c.1041G > A variation is located at the junction of exon 10 and intron 10, potentially affecting splicing and causing disease. Mutation taster (Disease_causing, predicted value of 1.0) and CADD software (predicted value of 33) predicted it as harmful. The SSP score was 0.93, indicating a high probability of splicing site activation. PCR electrophoresis showed that the amplified product of *ERCC8* cDNA in the subjects was about 198 bp shorter than the 716 bp product of the normal control, matching the length of exon 10. RNA high-throughput sequencing suggested significant absent of exon 10 at the transcriptional level. Therefore, we believe that the c.1041G> A synonymous variation affects splicing, resulting in exon 10 deletion. According to the ACMG guidelines, c.1041G > A (p. 282_347del) (PVS1 + PM2_supporting + PM3 + PP4) was upgraded to a pathogenic variation, suggesting that even synonymous mutations with unknown significance can be highly pathogenic.

It is worth noting that transcriptome sequencing *ERCC8* exon 1 did not reveal a deletion. Combined with the Quantitative real-time PCR, we believe that the exon 1 deletion may prevent the *ERCC8* allele from initiating transcription, thus failing to transcribe and reverse transcribe into cDNA for sequencing. In addition, in actual RNA high- throughput sequencing, exon 1 of *ERCC8* gene deletion can lead to undetectability of this strand due to abnormal transcription. Consequently, we speculate that only one strand of mRNA exists, producing one strand of cDNA for testing after reverse transcription. Therefore, the tested cDNA strand did not undergo deletion, showing no obvious deletion. Alternative splicing results indicated slightly lower mRNA expression of exon 1, but various factors affect mRNA expression.

In short, the patient ‘s two *ERCC8* alleles are affected as follows: one allele has exon 1 deletion preventing the initiation of the translation and transcription program, thus inhibiting normal protein expression; the other allele has a synonymous variation leding to the deletion of exon 10.

In this study, we used two different RNA sequencing methods and obtained consistent results: *ERCC8* exon 10 deletion. Transcriptome sequencing analyzes the total RNA transcribed in a specific functional state, including mRNA and non-coding RNA. It allows for the study of gene function and structure at an overall level without the need for pre-designed probes for known sequences. This method provides more accurate digital signals, higher detection throughput, and a wider detection range, though the error rate is relatively high. Therefore, despite RNA Sanger sequencing being cumbersome, time-consuming, with lower throughput, longer read lengths, and higher costs, it remains the gold standard for diagnosis. Consequently, we combined both methods to achieve the most accurate results.

At the same time, it should be noted that the patient’s disease is progressive, and the early stage was not diagnosed in time. The patient was admitted to the hospital at the age of 1 year and 7 months, with no obvious abnormalities in the head MRI results. At 3 years and 8 months, it was observed that white matter myelination was significantly delayed compared to children of the same age. By the age of 7 years, upon re-admission to the hospital, the patient had developed brain atrophy and low white matter mass. The final diagnosis was made based on comprehensive laboratory examination results and clinical phenotype parallel genetic diagnosis.

In 1970, Moosa and Dubowitz proposed that CS is a form of leukodystrophy [13]. Most subseqnent case reports supported their view. In 2010 [8], Koob conducted a retrospective analysis of 19 patients and summarized the main neuroimaging manifestations of CS disease as myelin dysplasia, calcification and brain atrophy. Early-onset patients exhibited more severe myelin dysplasia and significant calcification, while late-onset patients had less brain atrophy. These comprehensive neuroimaging findings are useful for the differential diagnosis of CS, distinguishing it from other childhood leukoencephalopathies and/or brain calcification. Baer et al. later screened 185 patient data from 135 articles published between 1988 and 2020 [1], finding that 67% of patients showed ventricular enlargement, 76% had white matter abnormalities, 57% had posterior fossa abnormalities, and 41% had cerebral calcification. Using “Cockayne Syndrome” and “*ERCC8*” as keywords, we retrieved 25 articles from 2020 to 2024, encompassing 33 cases. Among these, 79.2% of patients showed brain atrophy, 33.3% showed brain calcification, 25% had white matter abnormalities, and 37.5% had myelin abnormalities. In our case, the patient also exhibited brain atrophy, hypomyelination, and decreased white matter volume, consistent with previous research findings.

## Conclusions

In summary, as a rare neurodegenerative disease, patients with Cockayne syndrome (CS) often present with microcephaly, growth retardation, mental retardation, and white matter dysplasia. However, many of its molecular characteristics remain unclear, necessitating more clinical and molecular biological data to study their correlation further. Although heterozygous carriers show no obvious clinical manifestations, they risk passing on this harmful variation to their offspring. Therefore, a clear diagnosis of CS is essential for providing accurate prognostic guidance, genetic counseling, and prenatal diagnosis [5]. Currently, we diagnose CS primarily through clinical manifestations and imaging features, followed by molecular genetic diagnosis through genetic testing. Although synonymous mutations are rare, the incidence of disease at the junction of exons and introns is higher. We should be vigilant about this possibility to avoid misdiagnosis and missed diagnosis.

## Data Availability

Data sharing is not applicable to this article as no datasets were generated or analysed during the current study.
